# Handwriting or Typewriting? The Influence of Pen- or Keyboard-Based Writing Training on Reading and Writing Performance in Preschool Children

**DOI:** 10.5709/acp-0178-7

**Published:** 2015-12-31

**Authors:** Markus Kiefer, Stefanie Schuler, Carmen Mayer, Natalie M. Trumpp, Katrin Hille, Steffi Sachse

**Affiliations:** 1 Department of Psychiatry, Ulm University, Ulm, Germany; 2 ZNL Transfer Center for Neuroscience and Learning, Ulm University, Ulm, Germany; 3 Department of Developmental Psychology, University of Education Heidelberg, Heidelberg, Germany

**Keywords:** written language acquisition, literacy training, embodied cognition, digital media, preschool children

## Abstract

Digital writing devices associated with the use of computers, tablet PCs, or mobile phones are increasingly replacing writing by hand. It is, however, controversially discussed how writing modes influence reading and writing performance in children at the start of literacy. On the one hand, the easiness of typing on digital devices may accelerate reading and writing in young children, who have less developed sensory-motor skills. On the other hand, the meaningful coupling between action and perception during handwriting, which establishes sensory-motor memory traces, could facilitate written language acquisition. In order to decide between these theoretical alternatives, for the present study, we developed an intense training program for preschool children attending the German kindergarten with 16 training sessions. Using closely matched letter learning games, eight letters of the German alphabet were trained either by handwriting with a pen on a sheet of paper or by typing on a computer keyboard. Letter recognition, naming, and writing performance as well as word reading and writing performance were assessed. Results did not indicate a superiority of typing training over handwriting training in any of these tasks. In contrast, handwriting training was superior to typing training in word writing, and, as a tendency, in word reading. The results of our study, therefore, support theories of action-perception coupling assuming a facilitatory influence of sensory-motor representations established during handwriting on reading and writing.

## Introduction

Reading and writing are central cultural skills, which are typically acquired during childhood in societies with a strong literacy tradition. Mastering literacy is a key competence for success at school and in professional life (Gut, Reimann, & Grob, 2012). During the last years, the mode of writing in adults, but also in children has been subject of a dramatic change: Digital writing devices associated with the use of computers, tablet PCs, or mobile phones are increasingly replacing writing by hand (for overviews, see Mangen & Velay, 2010; Radesky, Schumacher, & Zuckerman, 2015). These changes of writing habits have a clear impact on basic sensory-motor skills: Compared with a high frequency of handwriting, in adults, a high frequency of keyboard use in producing written text in everyday life has been shown to be related to a decrement of the skill to produce precisely controlled arm-hand movements (Sulzenbrück, Hegele, Heuer, & Rinkenauer, 2010; Sulzenbrück, Hegele, Rinkenauer, & Heuer, 2011). The modulatory influence of writing habits on linguistic or cognitive performance, such as reading and writing, is less obvious, but it may represent an important factor to consider for identifying the optimal conditions for literacy training (Kiefer & Trumpp, 2012). In line with this reasoning, a recent survey among German teachers indicated that during the last years sensory-motor skills required for handwriting deteriorated among young children entering elementary school (Deutscher Lehrerverband, 2015). Given that children in our present days may get the first everyday writing experiences by typing on a computer or mobile phone much before they master handwriting (Mangen & Velay, 2010), it is important to know how this dramatic change in writing habits in the digital age affects written language acquisition. Addressing this issue is highly important for education because there is an increasing trend to introduce digital devices to kindergarten and elementary school (Herzig & Grafe, 2006). In some countries’ programs for elementary school education typing on digital devices has already replaced handwriting (Spitzer, 2015).

Regarding the influence of these modes of written language acquisition, handwriting versus typing, two competing theoretical approaches are possible. The motor program associated with typing is obviously easier than that associated with handwriting. Even small children intuitively interact with digital devices by typing or touching (Buchegger, 2013; Couse & Chen, 2010). This easiness of typing on digital devices is taken as an argument in favor of writing training with typing to accelerate writing in young children or in children with less developed sensory-motor skills (Calhoun, 1985; Castles et al., 2013; Doughty, Bouck, Bassette, Szwed, & Flanagan, 2013; Zheng, Warschauer, & Farkas, 2013). Such a view based on the easiness of motor programs associated with writing would predict better reading and writing performance when writing letters and words is trained by typing on a digital device. In support of this view, a small but positive correlation between frequency of computer use and letter knowledge has been found in a large cohort of four-year old children (Castles et al., 2013).

However, when comparing handwriting with typing, not only the easiness of the motor programs, but also their quality and the associated sensory-motor experiences (haptic, motor, visual etc.) must be considered. With respect to quality, handwriting and typing have fundamentally different properties (Mangen & Velay, 2010): Handwriting requires carefully reproducing the shape of each letter, whereas in typewriting the motor program is not related to the letter shape and, as a result, no such grapho-motor component is present. Hence, motor programs associated with handwriting provide an additional informative memory trace and may contribute to the representation of the shape of a letter (James & Engelhardt, 2012).

Such interactions between action and perception are important elements of embodied or grounded cognition theories, which state that cognition is essentially grounded in modality-specific sensory and motor systems (Barsalou, Simmons, Barbey, & Wilson, 2003; Gallese & Lakoff, 2005; Kiefer & Pulvermüller, 2012; Lakoff & Johnson, 1999; Pulvermüller, 2005). These theories assume that depending on the specific sensory-motor experience learning establishes memory traces, which are partially reactivated during retrieval. Interactions between action and perception are also predicted by the theory of event coding (Hommel, Muesseler, Aschersleben, & Prinz, 2001). According to this theory, perceptual contents and action plans are coded in a common representational medium by feature codes with distal reference. Perceptions and actions are proposed to be equally represented by integrated, task-tuned networks of feature codes, called event codes. Hence, several theories in the field of cognition, perception and action predict a superiority of handwriting over typing with regard to the quality of visual processing subserving reading and writing.

In line with the notion of action-perception couplings, interactions between action and perception have meanwhile been consistently observed in the field of visual object recognition (Bub & Masson, 2010; Bub, Masson, & Bukach, 2003; Hommel et al., 2001; Müsseler, Wühr, Danielmeier, & Zysset, 2005). For instance, action representations have been shown to facilitate recognition of objects with similar action affordances (Helbig, Graf, & Kiefer, 2006; Kiefer, Sim, Helbig, & Graf, 2011; Sim, Helbig, Graf, & Kiefer, 2014). Furthermore, when participants have to acquire the names and the meaning of novel objects, performing a meaningful action towards an object during training facilitates learning compared with a meaningless pointing action (v. Soden-Fraunhofen, Sim, Liebich, Frank, & Kiefer, 2008; Kiefer, Sim, Liebich, Hauk, & Tanaka, 2007). These results suggest that sensory-motor experiences during training must be meaningfully related to the learning target to result in stronger sensory-motor memory traces that facilitate recognition performance.

It is conceivable that similar mechanisms of action-perception coupling also influence letter recognition, reading, and writing performance. In line with this suggestion, several training studies in preschool children and adults showed that handwriting training of new letters gave not only rise to better spelling accuracy (Cunningham & Stanovich, 1990), but also improved letter recognition in a subsequent test compared with typing training (Longcamp et al., 2008; Longcamp, Zerbato-Poudou, & Velay, 2005; Naka, 1998). This demonstrates that handwriting, which links rich sensory-motor representations to perceptual letter shapes, improves not only writing, but also reading performance compared with typewriting. In line with this interpretation, neuroimaging studies showed that visual recognition of familiar letters activated not only visual areas, but also motor regions of the brain (James & Gauthier, 2006; Longcamp, Anton, Roth, & Velay, 2003; Longcamp, Hlushchuk, & Hari, 2011). When novel letters were trained by handwriting, a similar activation pattern was observed, which was absent when these novel letters were trained by typing (James & Atwood, 2009; Longcamp et al., 2008). Furthermore, handwriting experience also seems to be necessary in children to develop the adult-like neuronal circuit of letter processing encompassing visual and motor areas of the brain (James & Engelhardt, 2012).

Although several lines of evidence seem to suggest a superiority of handwriting training over typing training on subsequent reading and writing performance in young children, earlier studies mainly investigated recognition of individual letters and not reading or writing (Longcamp et al., 2005; Naka, 1998). Furthermore, the few studies investigating differential effects at the word level observed mixed results: While in one study a superiority of handwriting over typing training on spelling performance was found (Cunningham & Stanovich, 1990), this effect was not replicated in two other studies (Ouellette & Tims, 2014; Vaughn, Schumm, & Gordon, 1992). The mixed results may arise from the relatively short training programs, with only a few trials, from the inclusion of children of different age and literacy status (preschool vs. elementary school children), and from different training materials (words, pseudowords) and spelling tests (writing vs. recognition memory).

In order to contribute to this debate, for the present study, we developed an intense training program for preschool children attending the German kindergarten, with 16 training sessions distributed over four weeks on four days per week. We trained preschool children and not elementary schoolchildren, in order to assess training effects without the influence of previous formal handwriting training as in schoolchildren. Using closely matched letter learning games, eight letters of the German alphabet were trained either by handwriting with a pen on a sheet of paper or by typing on a computer keyboard. The handwriting and typing training programs were administered to two separate samples of preschool children aged between 4 and 6 years (handwriting: *n* = 12; typing: *n* = 11) matched for age, gender, and phonological awareness as possibly confounding variables. Letter recognition, letter naming, and initial letter writing performance were assessed before and after training. Reading and writing performance of four-letter words, which could be formed from the trained eight letters, were tested only post-training. Both handwriting and typing training were conducted by experimenters in a separate and quiet room of the kindergarten, but as part of the regular kindergarten schedule to obtain a naturalistic learning environment. If the easiness of the motor program facilitates letter recognition, reading, and writing, typing training should be superior to handwriting training. In contrast, we assumed that a meaningful coupling between action and perception should facilitate literacy training. We, therefore, expected that handwriting training should be superior to typing training.

## Method

### Participants

Participants were 23 children (12 female) aged between 4 years and 10 months, and 6 years and 3 months (*M* = 5 years and 6 months, *SD* = 4 months) recruited from two kindergartens (kindergarten 1: *n* = 9; kindergarten 2: *n* = 14) in the area of Ulm, Germany. All children were healthy according to the parents’ reports. The entire sample was split in two matched groups, which were assigned to the handwriting (*n* = 12) and typing (*n* = 11) training conditions, respectively. In each kindergarten, handwriting and typing training was conducted in a comparable number of children assigned to small subgroups of four to seven children. Depending on the specific training game, training occurred individually or in the entire subgroup (see below). Demographic data for the handwriting and typing training groups are shown in Table 1. Groups did not differ with regard to age, *t*(21) = 0.056, *p* =.956, gender, χ^2^(1) = 0.048. *p* = .827, and phonological awareness, *t*(21) = 0.330, *p* =.745, according to Bielefeld Screening for Early Detection of Difficulties in Reading and Writing (BISC) (Jansen, Mannhaupt, Marx, & Skowronek, 2002). The following BISC subscales contribute to the phonological awareness score: Rhyming, segmenting syllables, associating sounds, and relating sounds to words. Prior to the study, written informed consent was obtained from the parents of the children. The study was carried out according to the tenets of the Declaration of Helsinki.

**Table 1.** Demographic Data of the Children in the Typing and Handwriting Training Groups

	Training condition				
	Typing (*n* = 11)		Handwriting (*n* = 12)		Differences between groups
	Mean (*SD*)	Min-Max	Mean (*SD*)	Min-Max	
Age (in month)	66,1 (4,7)	58-75	66,0 (3,1)	61-72	*t*(21) = 0,056, *p* = .956
Phonological awareness (BISC score)	30,8 (4,2)	23-38	30,3 (4,1)	23-37	*t*(21) = 0,330, *p* = .745
Training attendance (in days)	12,9 (2,9)	8-16	14,1 (2,2)	9-16	*t*(21) = 1,106, *p* = .281
	Number females		Number females		
Gender	6		6		χ^2^(1) = 0.048, *p* = .827

*Note*. *SD* = standard deviation (values in parentheses).

### Letter training

**Figure 1.**
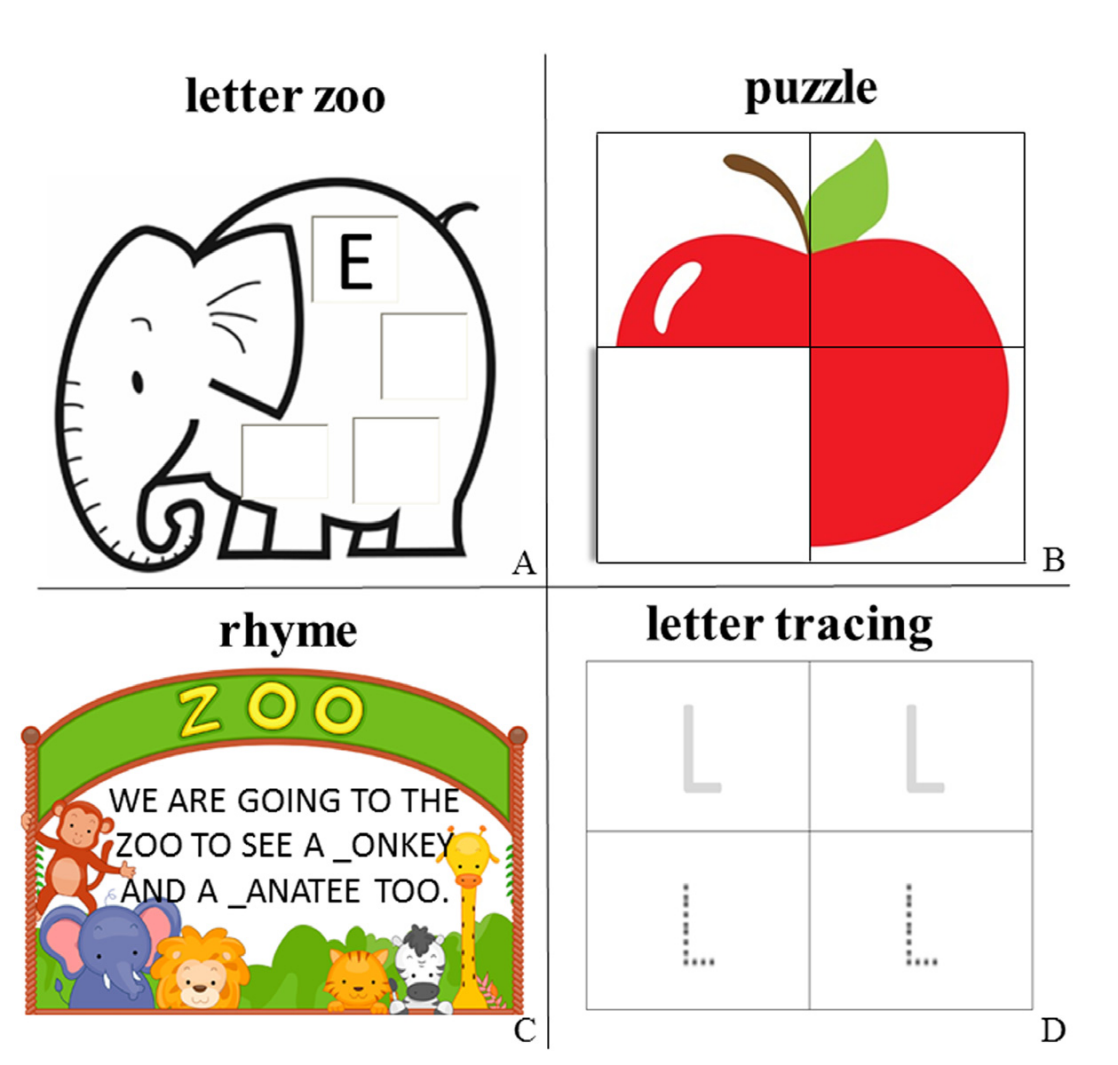
Overview of the training tasks used for written language training in preschool children. The same tasks were applied for typing and handwriting training. They differed only with regard to the writing mode (typing on a laptop keyboard vs. handwriting on a sheet of paper) in both training conditions. All texts were originally in German, shown are comparable examples in English translations.

Across four weeks, eight letters of the German alphabet (*L*, *I*, *O*, *A*, *M*, *S*, *T*, and *E*) were trained with letter games adopted from a German school booklet on literacy training (Reddig-Korn, Fritz, Mai, & Schmitt, 2003). Training procedure was identical for both the handwriting and typing program, except for the writing medium. In the handwriting training program, children wrote the letter with a pen on a sheet of paper. In the typing program, children typed the letter on a notebook keyboard, where only the keys with letters were visible (and additionally one key for navigation from one task to another), while the other keys were covered. Training sessions lasted about 25 min and took place on four days in each week resulting in a total of 16 sessions. Each week, two new letters were trained. On day one of each week, the first new letter was introduced, on day two the second one. In both training groups, the new letters were introduced to the children using a short story (adapted from Reddig-Korn et al., 2003). Children were told that two friends, Lili and Oli, are travelling to the Magic Letter Land, where they encounter new letters. The experimenter demonstrated not only the visual shape of the letter to the children, but also the corresponding sound and the lip movements used to produce the sound. Furthermore, the experimenter encouraged the children to search for words, which start with this letter. After this general introduction, children were trained individually on days one and two with the four letter learning games described below (see also Figure 1).

#### Letter zoo

Children learned to associate animal pictures with the corresponding initial letter of the animal names (e.g., *elephant*, and the letter *E*). Children were then presented with three pictures of animals and had to write or to type four times the initial letter of the animal name on each picture (Figure 1A). In the handwriting training program, pictures were printed on a sheet of paper, and the children could write the initial letter with a pen on the animal picture in four boxes within the picture. In the typing training program, each picture was presented in the middle of the notebook screen, and the initial letter of the animal name was typed in four boxes within the picture.

#### Puzzle

The children received a puzzle consisting of four parts, either on a sheet of paper or on a notebook screen (Figure 1B). The puzzle showed objects starting with one of the letters to be trained. There were three different objects per letter. Initially, all parts of the puzzle were shown with their backside up. Children were instructed to write/type the letter to be trained on the backside of each part (e.g., A). The experimenter assessed the correctness of the responses by determining whether the children wrote the letter correctly (position and relation of the lines correspond to the intended letter) or whether they pressed the key corresponding to the letter to be trained. If the children were correct, they were allowed to turn the puzzle part either physically in the handwriting training program or digitally on the notebook screen in the typing training program. If they were not correct, they had one more trial, before the experimenter showed the correct answer. The children could then repeat the correct response.

#### Rhyme completion

The experimenter showed the children a sentence containing a pair of rhyming words either on a sheet of paper or on the computer screen (Figure 1C). The children were told that the letter to be trained (e.g., *M*) is missing once in each word. A gap indicated the missing letter. Depending on the training group, the children wrote/typed the missing letter in the gap. If the child inserted the letter correctly, the experimenter read the sentence with the rhyming words aloud. Each letter was trained once per session. The rhyme only had the function to motivate the child, but the writing/typing task was not related to the rhyme.

#### Letter tracing

Children received sheets of paper with the letter clearly printed above and printed with unconnected dots below (Figure 1D). In both groups, the children were instructed to recognize the letter printed in dots and to reproduce it either by handwriting or by typing: In the handwriting group, children had to trace the shape of the letter by connecting the dots using a pen. In the typing group, children had to find and to type the letter on the keyboard, which then appeared on the screen. Each letter was traced/typed twice.

Days three and four of each week served to repeat all trained letters with a variation of letter learning games to render the training more interesting for the children. In order to train reading and writing, on day three, the children received the letter zoo and the puzzle games, and, as a novel aspect, the letter recognition as well as the letter and word writing tasks (for a task description, see below). Children were again trained individually.

Training on day four of each week occurred within small groups of about four children and was based on a new set of training games: All letters introduced so far were trained on day four in a more informal play situation using the following letter games.

#### Gremlin

In the middle of the table, there was a deck with cards displaying objects that started with the letters to be trained. The children of the group took turns throwing a special dice, which had three possible outcomes: If the dice showed a flower, the depicted object should be named and the initial letter should be written on a paper/typed on the keyboard. As reward, the child was allowed to place the card in a “collecting pot”. If the dice showed the gremlin, the gremlin “took the card away”. If the dice showed the fairy, the fairy took the card from the gremlin and placed it back in the middle of the table. Children were instructed to collect as many cards as possible in the pot for the group, otherwise the gremlin would win.

#### Letter ship

In the middle of the table, there was again a deck with cards displaying objects that started with the letters to be trained. There were three cards per letter. There was a letter ship, which was placed in the middle of the table. The children were told that this ship only carries objects starting with one specific letter. One child started and sequentially turned the cards from the deck. She or he had to decide whether the object depicted on each card started with the letter that is carried by the ship. If the response was correct, the child was allowed to write/type the letter on a small paper ship/empty text document and put the card in the big letter ship in the middle of the table. Then the next child took the turn. The laptop/paper was placed close to the cards and the letter ship in order to keep switching of attention comparable across groups. In the typing group, the typed letter appeared on the computer screen.

#### Magic potion

In the middle of the table, there was again a deck with cards displaying objects that started with the letters to be trained. There were two cards per letter. One child started, turned two cards from the deck and read the letters out loud. If the initial letters of the two depicted objects were identical, the child wrote/typed the letter on a response card. In the typing training group, the “response card” was an empty computer screen. The letter could then be used as an “ingredient” for the magic potion brewed by a witch. Otherwise, the cards were placed back on the bottom of the deck. Then the next child took the turn. If all matching cards were taken away, the “magic potion” was ready.

An overview of the learning games used on training days one to four is given in Table 2. Training in weeks one to four was comparable, except for the introduction of new letters and for the repetition of an increasing number of letters (week 1: two letters, week 2: four letters, week 3: six letters, week 4: eight letters). During training, the children received feedback regarding the correctness of their response. All training and testing tasks (for the latter, see below) were conducted by one of the two experimenters. Both experimenters were responsible for training and testing of a comparable number of participants in both the handwriting and typewriting training groups (experimenter 1: 7/7, experimenter 2: 5/4).

**Table 2.** Assignment of Training Tasks to Training Days in Each Week

	Letter tracing	Letter zoo	Rhyme completion	Puzzle	Letter writing	Word writing	Letter recognition
Day 1	X	X	X	X			
Day 2	X	X	X	X			
Day 3		X		X	X	X	X
Day 4	week 1 and 2: letter ship, gremlin week 3 and 4: magic potion						

### Test tasks on letter recognition, reading, and writing

#### Letter recognition

Each child was presented with a card showing one of the eight letters to be learnt among three visually similar pseudoletters. The task was to select the real letter among the distractors. The dependent measure in this task was the number of correct recognition responses of the eight trained letters (0-8 letters). This task was administered before and after the training as test task as well as during the training sessions at day three within each week as training task.

#### Letter naming

Each child was sequentially shown all 26 letters of the alphabet on a card ordered by difficulty (according to Reddig-Korn et al., 2003). The child was instructed to say “stop”, when the letter was familiar, and asked to name the letter. The dependent measure in this task was number of correct reading responses of the eight trained letters (0-8 letters). This task was administered before and after training.

#### Word reading

Each child received cards with the words *OMI* (Eng.: *grandma*), *TAL* (Eng.: *valley*), *TESA* (Eng.: *tape*), which were formed from the trained letters. The child was told to read each word aloud. Dependent measure was the number of correctly read words (0-3 words). A word was considered to be correctly read, when the pronounced phonemes corresponded to the target word. This task was only administered after the training.

#### Letter writing

The experimenter sequentially read the trained letters to the child aloud. Each of the letters was read aloud twice in random order (*L*, *T*, *S*, *I*, *A*, *O*, *T*, *M*, *I*, *E*, *O*, *L*, *M*, *S*, *E*, *A*). The child was instructed to write down the letter on a sheet of paper or to type it on the keyboard depending on the training program. Dependent measure was number of correctly written/typed letters (0-16 letters). The experimenter assessed the correctness of the responses by determining whether the children wrote the letter correctly (position and relation of the lines correspond to the intended letter) or pressed the key corresponding to the target letter. This task was only administered after the training. On day three of each training week, this task was also presented to the children as training task. When used as training task, only the two letters trained in the corresponding week were presented, while each letter was read aloud four times in a random order.

#### Free letter writing

Each child was instructed to write with a pen all familiar letters on a sheet of paper. In this task, only the trained eight letters were analyzed. Dependent measure was the number of correctly written letters (0-8 letters). A letter was considered to be correctly written, when the position and relation of the lines corresponded to the intended target letter. Children received this task before and after training. This task was administered as handwriting version only because it also served to assess letter writing performance before training. Of course, possible differential training effects between groups cannot be unequivocally interpreted in this task because in the typing group writing mode at test differed from that at training.

#### Word writing

The experimenter read the four words *LILI* (Eng.: the name *Lili*), *OLI* (Eng.: the name *Oli*), *SALAMI* (Eng.: *salami*), *TASTE* (Eng.: *key*) aloud at a slow pace. The child was told to write or to type the word depending on the training program. Depending variable was percentage of correctly written/typed letters independent of their position (0-100%). This task was administered after the training. During training on day three, this task was also presented as training task to the children, but only with words that could be formed from the letters trained so far.

## Results

### Training attendance

The children attended on average 13.5 of the 16 training sessions (*SD* = 2.56; min: 8; max: 16). Children missed sessions due to absence from kindergarten (e.g., vacation or illness). Handwriting and typing training groups did not differ with regard to attendance, handwriting: *M* = 14.1, *SD* = 2.2, range 9-16; typing: *M* = 12.9, *SD* = 2.9, range 8-16; *t*(21) = 1.106, *p* = .281. As the number of attended sessions did not correlate with outcome measures of the training, all children were included in the final analysis irrespective of frequency of attendance.

### Letter recognition, reading, and writing performance

**Figure 2.**
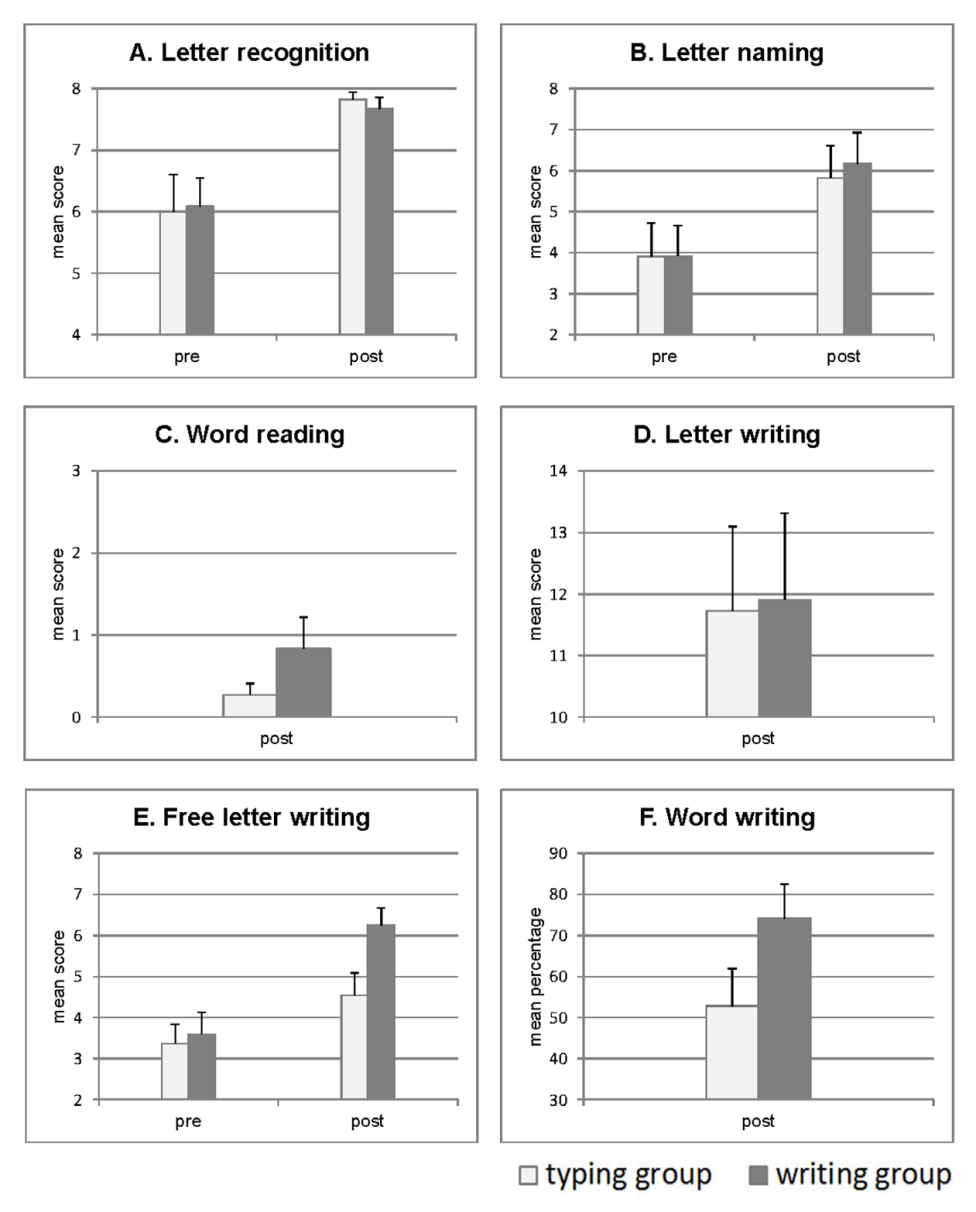
Letter recognition, reading, and writing performance of the preschool children in the typing versus handwriting training conditions. Shown are mean scores (number of correct responses) or mean percentage scores (relative frequency of correct responses).

An overview of the test results as a function of training mode (typing vs. handwriting) is given in Table 3 and Figure 2. Please note that some tests were only administered after training. When tests were administered before and after the training, we first performed repeated-measures analyses of variance (ANOVA) with the factor training (before and after training) as within-subject factor and the factor group (typing vs. handwriting) as between-subjects factor. Training effects within groups (performance after vs. before training) and between groups (comparison of gain scores calculated as performance difference after minus before training) were analyzed in more detail using one-tailed *t*-tests for dependent and independent samples, respectively. Data of tests that were only administered after the training were compared between groups using one-tailed *t*-tests for independent samples. In order to control for possible performance differences between groups before training, two-tailed *t*-tests for independent samples were performed.

**Table 3.** Overview of Letter Recognition, Reading, and Writing Performance of the Children as a Function of Typing Versus Handwriting Training

	Training condition						
	Typing (*n* = 11)			Handwriting (*n* = 12)			Differences between groups
	Pre	Post	Difference Pre - Post	Pre	Post	Difference Pre - Post	
	Mean (*SD*)[Min-Max]	Mean (*SD*)[Min-Max]		Mean (*SD*)[Min-Max]	Mean (*SD*)[Min-Max]		
Letter recognition	6,00 (2,00) [1-8]	7,82 (0,40) [7-8]	*p* = .006	6,08 (1,62) [4-8]	7,67 (0,65) [6-8]	*p* = .006	*p* = .384
Letter naming	3,91 (2,70) [0-7]	5,82 (2,60) [1-8]	*p* < .001	3,92 (2,61) [0-8]	6,17 (2,62) [1-8]	*p* = .004	*p* = .674
Word reading	---	0,27 (0,47) [0-1]	---	---	0,83 (1,34) [0-3]	---	*p* = .097
Letter writing	---	11,73 (4,54) [3-16]	---	---	11,92 (4,83) [2-16]	---	*p* = .462
Free letter writing	3,36 (1,57) [0-5]	4,55 (1,81) [1-7]	*p* = .05	3,58 (1,88) [1-8]	6,25 (1,42) [4-8]	*p* <.001	*p* = .047, *d* = 0,63
Word writing	---	52.84 (30.05) [18.25-100]	---	---	74.17 (28.60) [10-100]	---	*p* = .048, *d* = 0.76

*Note*. **SD** = standard deviation (values in parentheses).

#### Letter recognition (Figure 2A)

The ANOVA only revealed a significant main effect of training, *F*(1, 21) = 18.7, *p* < .0003. Subsequent *t*-tests confirmed that letter recognition did not differ between handwriting and typing groups before training, *t*(21) = 0.110, *p* = .913. Both groups showed increased letter recognition performance after compared with before training, writing group: *t*(11) = 2.994, *p* = .006; typing group: *t*(10) = 3.108, *p* = .006, but this training effect did not differ between groups, *t*(21) = 0.299, *p* = .384.

#### Letter naming (Figure 2B)

The ANOVA only yielded a significant main effect of training, *F*(1, 21) = 27.02, *p* < .0001. Subsequent *t*-tests again confirmed that letter naming performance did not differ between groups before training, *t*(21) = 0.007, *p* =.995. Training increased letter naming performance, writing group: *t*(11) = 3.276, *p* = .004; typing group: *t*(10) = 5.186, *p* < .001, in each group, but this increment did not differ between groups, *t*(21) = 0.426, *p* = .674.

#### Word reading (Figure 2C)

Word reading was only assessed after training. There was a tendency for superior reading performance in the handwriting group compared with the typing group, *t*(14) = 1.364, *p* = .097. Note that accuracy distribution in the handwriting group ranged from zero to perfect performance (all three words named correctly) and was much larger than accuracy distribution in the typing group, which varied between zero and one correct response. Due to this unequal variance in both groups, degrees of freedoms had to be adjusted.

#### Letter writing (Figure 2D)

Letter writing carried out either by handwriting or typing did not differ between groups, *t*(21) = 0.097, *p* = .462. This test was only administered after training.

#### Free letter writing (Figure 2E)

The ANOVA yielded a significant main effect of training, *F*(1, 21) = 20.68, *p* < .0002, as well as a trend for an interaction between group and training, *F*(1, 21) = 3.08, p < .09. Subsequent two-tailed *t*-tests for independent samples showed that before training handwriting and typing groups did not differ in free letter writing, *t*(21) = 0.303, *p* = .765. Training increased free letter writing performance in each group, *t*(11) = 4.927, *p* <.001; *t*(10) = 1.796, *p* = .05. Handwriting training resulted in a significantly greater increment of performance compared with typing training, *t*(21) = 1.76, *p* = .047, *d* = 0.63. Results obtained before training confirm that initial letter writing knowledge was comparable for the handwriting and typing groups. However, as differences between handwriting and typing training groups cannot be interpreted after training due to a differential match between training (handwriting vs. typing) and test mode (handwriting only), we do not further discuss findings of this task.

#### Word writing (Figure 2F)

Word writing carried out by handwriting was superior to word writing carried out by typing, *t*(21) = 1.744, *p* = .048, *d* = 0.76. This test was only administered after training.

#### Age effects on reading and writing performance

The age range among the trained children was about one year and half (4 years and 10 months, to 6 years and 3 months). In order to estimate how letter knowledge and writing performance was related to age, we correlated children’s age with test results assessed before training. As we wanted to obtain a reasonable sample size, we performed this analysis for all children pooled across training groups for the pre-training data only. Using Spearman’s rank correlation, we found a significant positive correlation between age and letter recognition performance (*r*_s_ = .548; *p* = .025, *N* = 23). However, age was not related to word reading and writing performance. Hence, despite some differences in single letter knowledge, initial reading and writing performance was relatively comparable within the studied age range before training.

## Discussion

The present study investigated the influence of two modes of written language training on letter recognition, reading and writing performance in matched groups of preschool children: In one group of children eight letters were trained by writing them with a pen on a sheet of paper, whereas in the other group training involved typing the same set of letters on a computer keyboard. In other respects, training sessions and tasks for handwriting and typing training were designed as comparable as possible. If the easiness of the motor program facilitates letter recognition, reading and writing, a superiority of typing training over handwriting training should be found (Calhoun, 1985; Castles et al., 2013; Doughty et al., 2013; Zheng et al., 2013). In contrast, if a meaningful coupling between action and perception facilitates literacy training (Barsalou et al., 2003; Gallese & Lakoff, 2005; Hommel et al., 2001; Kiefer & Pulvermüller, 2012; Lakoff & Johnson, 1999; Pulvermüller, 2005), as we assumed, handwriting training should be superior to typing training.

Overall, the results of this study were relatively clear-cut. In none of the test tasks administered to the children after training, we found superior performance after typing training compared with handwriting training. Even in tasks such as single letter writing, in which the easier motor program associated with typing could be most advantageous, accuracy was not higher in the typing than in the handwriting training group. Instead, performance did not differ across groups. Thus, our results are entirely inconsistent with the notion that the easiness of the motor program associated with typing is beneficial for written language training, at least in children without disabilities.

However, results of this study at least partially support theories of action and perception coupling because superior accuracy for handwriting training was found in several word reading and writing tasks. We found superior word writing accuracy after handwriting training compared with typing training. This result replicates earlier work (Cunningham & Stanovich, 1990) and suggests that sensory-motor memory traces acquired during handwriting training support spelling of words, presumably due to improved memory for letters (Naka, 1998). These findings are particularly remarkable because children wrote the words using the trained writing method—that is, handwriting versus typing letters on keyboard. Thus, our findings in the word writing test cannot be explained by a change of the writing mode between training and test in the typing training group. Unlike the present findings and those by Cunningham and Stanovich (1990), other studies found comparable writing performance after handwriting and typing training (Ouellette & Tims, 2014; Vaughn et al., 1992). Presumably, the divergent results can be explained by the different length of the training program (16 days as in our study vs. one or a few days), age and literacy status of the children (largely preliterate preschool children as in our study vs. elementary school children), training materials (words vs. pseudowords) or test tasks (writing/typing as in our study or multiple choice recognition memory test). We assume that in laboratory studies superior writing performance after handwriting training can only be obtained when training is sufficiently long to establish enduring sensory-motor memory traces. Furthermore, unlike in preschool children differential training effects might be masked in schoolchildren, who have already received a substantial amount of written language training using handwriting. Finally, a multiple choice recognition memory test, in which performance is based on stimulus familiarity to a large degree (Jacoby, 1991), might be not sensitive enough to measure possibly beneficial effects of sensory-motor memory traces established during handwriting. Word reading accuracy tended to be higher in the handwriting group, although this difference was not statistically significant, presumably due to the small sample size and the relatively large variability in the handwriting group. Nevertheless, in line with theories of perception-action coupling (Barsalou et al., 2003; Gallese & Lakoff, 2005; Hommel et al., 2001; Kiefer & Pulvermüller, 2012; Lakoff & Johnson, 1999; Pulvermüller, 2005), this observation suggests that the motor program associated with handwriting facilitates word recognition compared with typewriting.

In contrast to our expectations and to previous findings (Longcamp et al., 2005, 2008; Naka, 1998), handwriting training did not improve letter recognition and letter naming performance compared with typing training. Overall, letter recognition performance before training was relatively high even in preschool children, presumably because children were already familiarized with some letters earlier in their lives. Pre-experimental letter knowledge was in particular high for the older children, as demonstrated by the positive correlation between letter recognition performance before training and age. After training, children in both groups performed close to ceiling in both letter recognition and letter naming. This reduces the likelihood to observe differential effects of the two training regimens.

Interpretation of the results is limited by the small sample size in both training groups, which reduces the statistical power to detect effects. The small sample size also precludes the assessment of age effects on training efficacy: It is possible that older children (> 6 years) benefit more strongly from handwriting training due to superior hand motor skills compared with younger children (< 5 years) leading to stronger training effects on reading and writing performance in this age group (cf. Longcamp et al., 2005). However, age effects on handwriting training remain to be addressed in future studies. Furthermore, only eight letters where trained, which led to ceiling effects for letter recognition and letter reading. Finally, performance for the word reading and writing tasks was generally quite low indicating that even a training over six weeks is not sufficient to obtain a high performance level in preschool children, who are at the beginning of written language acquisition. Please note however that our training regimen already involved many more training sessions compared with earlier work, in which no differential effects of training modes were reported (Ouellette & Tims, 2014; Vaughn et al., 1992). Furthermore, potential variation in the dependent measures of the word reading and writing task was quite low (0-3 words) because only a few words could be formed from the small number of trained letters. Future work should therefore replicate the present work with a larger sample of children and more letter stimuli for a more extended time.

Despite these limitations, the present study demonstrates that training studies in preschool children are a promising way to study modes of literacy training within naturalistic kindergarten settings. Our work clearly demonstrates that the easiness of the motor program associated with typing on digital devices does not facilitate written language acquisition compared with handwriting training: In none of our test tasks, children of the typing training group showed superior letter recognition, reading, or writing performance compared with children who received writing training based on handwriting. Of course, our results do not preclude the possibility that typing on digital devices might be useful to support writing in children with motor impairments that affect handwriting. Most importantly, we found that children of the handwriting training group performed better than those of the typing group particularly in tasks involving reading and writing at the word level. Our results, therefore, support theories of action-perception coupling assuming a facilitatory influence of sensory-motor representations established during handwriting on reading and writing performance.
